# Progressive thalamic nuclear atrophy in blepharospasm and blepharospasm-oromandibular dystonia

**DOI:** 10.1093/braincomms/fcae117

**Published:** 2024-04-08

**Authors:** Jinping Xu, Yuhan Luo, Jiana Zhang, Linchang Zhong, Huiming Liu, Ai Weng, Zhengkun Yang, Yue Zhang, Zilin Ou, Zhicong Yan, Qinxiu Cheng, Xinxin Fan, Xiaodong Zhang, Weixi Zhang, Qingmao Hu, Dong Liang, Kangqiang Peng, Gang Liu

**Affiliations:** Institute of Biomedical and Health Engineering, Shenzhen Institutes of Advanced Technology, Chinese Academy of Sciences, Shenzhen 518055, China; Department of Neurology, The First Affiliated Hospital, Sun Yat-Sen University, Guangdong Provincial Key Laboratory for Diagnosis and Treatment of Major Neurological Diseases, National Key Clinical Department and Key Discipline of Neurology, Guangzhou 510080, China; Department of Neurology, The First Affiliated Hospital, Sun Yat-Sen University, Guangdong Provincial Key Laboratory for Diagnosis and Treatment of Major Neurological Diseases, National Key Clinical Department and Key Discipline of Neurology, Guangzhou 510080, China; Department of Medical Imaging, Sun Yat-Sen University Cancer Center, State Key Laboratory of Oncology in Southern China, Collaborative Innovation Center for Cancer Medicine, Guangzhou 510060, China; Department of Medical Imaging, Sun Yat-Sen University Cancer Center, State Key Laboratory of Oncology in Southern China, Collaborative Innovation Center for Cancer Medicine, Guangzhou 510060, China; Department of Neurology, The First Affiliated Hospital, Sun Yat-Sen University, Guangdong Provincial Key Laboratory for Diagnosis and Treatment of Major Neurological Diseases, National Key Clinical Department and Key Discipline of Neurology, Guangzhou 510080, China; Department of Neurology, The First Affiliated Hospital, Sun Yat-Sen University, Guangdong Provincial Key Laboratory for Diagnosis and Treatment of Major Neurological Diseases, National Key Clinical Department and Key Discipline of Neurology, Guangzhou 510080, China; Department of Neurology, The First Affiliated Hospital, Sun Yat-Sen University, Guangdong Provincial Key Laboratory for Diagnosis and Treatment of Major Neurological Diseases, National Key Clinical Department and Key Discipline of Neurology, Guangzhou 510080, China; Department of Neurology, The First Affiliated Hospital, Sun Yat-Sen University, Guangdong Provincial Key Laboratory for Diagnosis and Treatment of Major Neurological Diseases, National Key Clinical Department and Key Discipline of Neurology, Guangzhou 510080, China; Department of Neurology, The First Affiliated Hospital, Sun Yat-Sen University, Guangdong Provincial Key Laboratory for Diagnosis and Treatment of Major Neurological Diseases, National Key Clinical Department and Key Discipline of Neurology, Guangzhou 510080, China; Institute of Biomedical and Health Engineering, Shenzhen Institutes of Advanced Technology, Chinese Academy of Sciences, Shenzhen 518055, China; Institute of Biomedical and Health Engineering, Shenzhen Institutes of Advanced Technology, Chinese Academy of Sciences, Shenzhen 518055, China; Institute of Biomedical and Health Engineering, Shenzhen Institutes of Advanced Technology, Chinese Academy of Sciences, Shenzhen 518055, China; Department of Neurology, The First Affiliated Hospital, Sun Yat-Sen University, Guangdong Provincial Key Laboratory for Diagnosis and Treatment of Major Neurological Diseases, National Key Clinical Department and Key Discipline of Neurology, Guangzhou 510080, China; Institute of Biomedical and Health Engineering, Shenzhen Institutes of Advanced Technology, Chinese Academy of Sciences, Shenzhen 518055, China; Institute of Biomedical and Health Engineering, Shenzhen Institutes of Advanced Technology, Chinese Academy of Sciences, Shenzhen 518055, China; Department of Medical Imaging, Sun Yat-Sen University Cancer Center, State Key Laboratory of Oncology in Southern China, Collaborative Innovation Center for Cancer Medicine, Guangzhou 510060, China; Department of Neurology, The First Affiliated Hospital, Sun Yat-Sen University, Guangdong Provincial Key Laboratory for Diagnosis and Treatment of Major Neurological Diseases, National Key Clinical Department and Key Discipline of Neurology, Guangzhou 510080, China

**Keywords:** blepharospasm-oromandibular dystonia, causal effects, deep brain stimulation, neuromodulation, thalamic nuclei

## Abstract

The thalamus is considered a key region in the neuromechanisms of blepharospasm. However, previous studies considered it as a single, homogeneous structure, disregarding potentially useful information about distinct thalamic nuclei. Herein, we aimed to examine (i) whether grey matter volume differs across thalamic subregions/nuclei in patients with blepharospasm and blepharospasm-oromandibular dystonia; (ii) causal relationships among abnormal thalamic nuclei; and (iii) whether these abnormal features can be used as neuroimaging biomarkers to distinguish patients with blepharospasm from blepharospasm-oromandibular dystonia and those with dystonia from healthy controls. Structural MRI data were collected from 56 patients with blepharospasm, 20 with blepharospasm-oromandibular dystonia and 58 healthy controls. Differences in thalamic nuclei volumes between groups and their relationships to clinical information were analysed in patients with dystonia. Granger causality analysis was employed to explore the causal effects among abnormal thalamic nuclei. Support vector machines were used to test whether these abnormal features could distinguish patients with different forms of dystonia and those with dystonia from healthy controls.

Compared with healthy controls, patients with blepharospasm exhibited reduced grey matter volume in the lateral geniculate and pulvinar inferior nuclei, whereas those with blepharospasm-oromandibular dystonia showed decreased grey matter volume in the ventral anterior and ventral lateral anterior nuclei. Atrophy in the pulvinar inferior nucleus in blepharospasm patients and in the ventral lateral anterior nucleus in blepharospasm-oromandibular dystonia patients was negatively correlated with clinical severity and disease duration, respectively. The proposed machine learning scheme yielded a high accuracy in distinguishing blepharospasm patients from healthy controls (accuracy: 0.89), blepharospasm-oromandibular dystonia patients from healthy controls (accuracy: 0.82) and blepharospasm from blepharospasm-oromandibular dystonia patients (accuracy: 0.94). Most importantly, Granger causality analysis revealed that a progressive driving pathway from pulvinar inferior nuclear atrophy extends to lateral geniculate nuclear atrophy and then to ventral lateral anterior nuclear atrophy with increasing clinical severity in patients with blepharospasm. These findings suggest that the pulvinar inferior nucleus in the thalamus is the focal origin of blepharospasm, extending to pulvinar inferior nuclear atrophy and subsequently extending to the ventral lateral anterior nucleus causing involuntary lower facial and masticatory movements known as blepharospasm-oromandibular dystonia. Moreover, our results also provide potential targets for neuromodulation especially deep brain stimulation in patients with blepharospasm and blepharospasm-oromandibular dystonia.

## Teaser

Xu *et al*. have studied the differences in thalamic nuclei volumes between blepharospasm and blepharospasm-oromandibular dystonia, further explored the causal effects among abnormal thalamic nuclei and concluded that thalamic nuclear atrophy is the important underlying neuromechanism in patients with blepharospasm and blepharospasm-oromandibular dystonia.

## Graphical Abstract

**Graphical Abstract:**
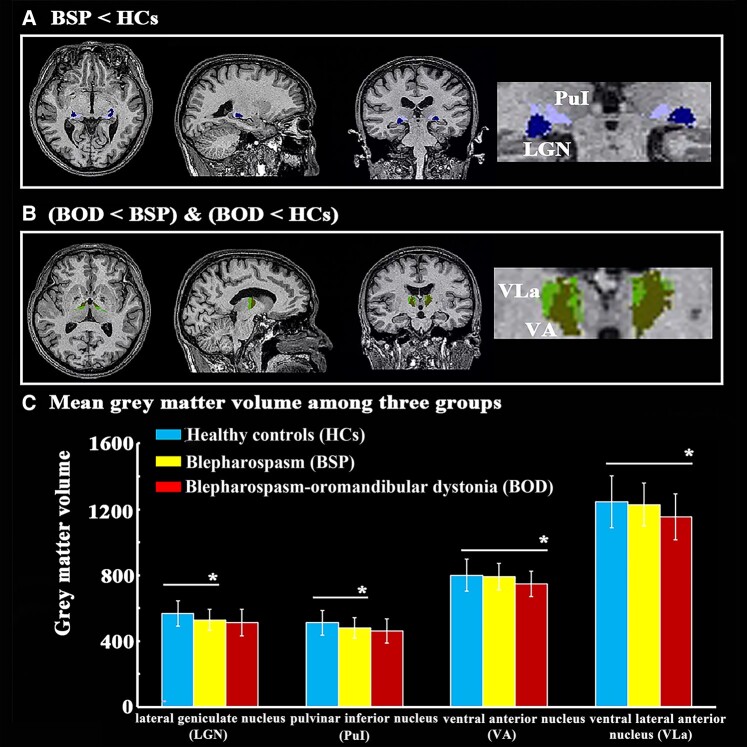

## Introduction

Blepharospasm (BSP), a focal dystonia characterized by excessive blinking and eyelid spasms, can lead to functional blindness in its most severe form.^1^ Patients with BSP also have the highest risk and fastest spread rate of symptoms to other body regions, most commonly to the oromandibular region.^2^ They can be associated with complex movements of the lower facial muscles, mouth, jaw and tongue. However, the neural mechanisms underlying the spread of symptoms in patients with BSP are poorly understood.

Current neurophysiological and neuroimaging evidence^3^ suggests a network model in which various brain regions play a role in the BSP pathogenesis, including the brainstem nuclei, basal ganglia, thalamus, cerebellum and cerebral cortex. Among these, the thalamus is suggested to be a key integrative hub in this network, receiving and distributing information among different brain regions.^4^ Lesion studies have shown that thalamic lesions are prone to induce BSP,^5,6^ further emphasizing the potential key role of the thalamus in the pathogenesis of BSP. Previous functional neuroimaging studies on patients with idiopathic BSP have revealed increased cortical activation,^7,8^ glucose hyper-metabolism,^9,10^ decreased amplitude of low-frequency fluctuations,^11,12^ decreased functional connectivity profiles in the thalamus^13,14^ and abnormal cortico-thalamic circuits.^15,16^ Structural neuroimaging studies also revealed commonly changed fractional anisotropy and mean diffusivity in white matter related to the thalamus,^17-19^ as well as decreased thalamic volume in idiopathic BSP.^20^ In contrast, only one study has reported functional changes,^21^ and two have reported white matter damage^18,19^ in the thalamus in patients with blepharospasm-oromandibular dystonia (BOD). A previous voxel-based morphological study involving 46 patients with BOD showed a decrease in the volume of multiple cerebral cortical regions without thalamic involvement, suggesting that patients with BOD may have abnormal integration at the cortical but not cortical–subcortical level.^19^ However, thalamus was considered as a single, homogeneous structure in most previous studies, disregarding potentially useful information about distinct thalamic nuclei. The thalamus contains distinct nuclei serving different functions, which may therefore be related to different symptoms or disorders.^22,23^ Recently, a statistical atlas of the thalamus was constructed using ultra-high-resolution *ex vivo* MRI combined with *in vivo* data (available in FreeSurfer 7, https://surfer.nmr.mgh.harvard.edu/fswiki/rel7downloads).^24^ This atlas showed a good agreement with previous histological studies of the thalamus in terms of volumes of representative nuclei and exhibited excellent test–retest reliability and robustness to changes in input MRI contrast. Moreover, it has been used to detect different thalamic effects in many disorders such as Alzheimer’s disease,^24^ schizophrenia^25^ and Parkinson’s disease.^26^ Although these thalamic atlases were published many years ago, fine thalamic nuclei in dystonia have yet to be investigated.

In the present study, we adopted this ideal atlas to investigate whether and how thalamic nuclear atrophy occurs in BSP and/or BOD for the first time. We collected structural MRI data from 56 patients with idiopathic BSP, 20 with BOD and 58 healthy controls (HCs) to explore (i) whether grey matter volume differed across thalamic subregions/nuclei in BSP and BOD, and whether such alteration was shared or specific to BSP or BOD; (ii) whether causal effects or pathways existed among abnormal thalamic nuclei; and (iii) whether these abnormal thalamic nuclei could be used as neuroimaging biomarkers to distinguish BSP from BOD patients and patients with dystonia from HCs.

## Materials and methods

### Participants

Patients diagnosed with adult-onset BSP and BOD were recruited from our outpatient clinic for movement disorders. The patients were diagnosed by senior neurologists (G.L. and Z.O.) based on published standard criteria.^27-29^ Exclusion criteria included the following: (i) botulinum toxin (BoNT) injections within 3 months prior to imaging; (ii) evidence of traumatic brain injury, stroke, Alzheimer’s disease, Parkinson’s disease or epilepsy; (iii) exposure to antipsychotic medications prior to the onset of dystonia; (iv) family history of movement disorders; and (v) had medical implants, which was a contraindication for cerebral MRI. Moreover, we only included BOD patients initially presenting with BSP. Finally, 56 patients with idiopathic BSP, 20 with BOD and 58 HCs were included.

### Patient consent

All participants provided written informed consent according to the Declaration of Helsinki, and the study was approved by the ethics committee of the First Affiliated Hospital of Sun Yat-Sen University ([2020]323).

### Clinical assessment

Patient demographics and clinical characteristics, including age, sex, duration of disease and BoNT injections, were obtained during face-to-face interviews prior to MRI. The Jankovic Rating Scale (JRS)^30^ was used to assess the severity of BSP, and the Burke-Fahn-Marsden Dystonia Rating Scale (BFMDRS)^31^ was used to assess the severity of BOD.

### Image acquisition

3D T_1_-weighted data were collected using a 3T MRI scanner (Tim Trio; Siemens, Erlangen, Germany) with a magnetization-prepared rapid acquisition gradient-echo pulse sequence. The main parameters were as follows: repetition time = 2530 ms; echo time = 4.45 ms; inversion time = 1100 ms; flip angle = 7^○^; matrix dimensions = 256 mm × 256 mm; voxel size = 1 × 1 × 1 mm^3^; and 192 slices.

### Image preprocessing

All T_1_ images were processed using the standard segmentation pipeline available in the FreeSurfer v7.1.1 by using default settings. The main steps included skull stripping, Talairach registration and initialization of cortical surface reconstruction, cortical atlas registration and subcortical parcellation. The estimated total intracranial volume was also calculated. To quantify thalamic nuclear volume, we implemented automatic parcellation of 25 thalamic nuclei in each hemisphere based on manual delineation combining *in vivo* and *ex vivo* data.^24^ Visualized inspection confirmed that the automatic segmentation and labelling were performed accurately. Because some of the thalamic nuclei are relatively small with mean grey matter volume < 200 mm^3^, we included only 15 thalamic nuclei in the further analysis. These nuclei included one nucleus in the anterior group (anteroventral), one in the lateral group (lateral posterior), four in the ventral group (ventral anterior, ventral lateral anterior, ventral lateral posterior and ventral posterolateral), one in the intralaminar group (centromedian), two in the medial group (mediodorsal medial magnocellular and mediodorsal lateral parvocellular) and six in the posterior group (lateral geniculate, medial geniculate, pulvinar anterior, pulvinar medial, pulvinar lateral and pulvinar inferior). Finally, Freeview (https://surfer.nmr.mgh.harvard.edu/fswiki/FreeviewGuide/FreeviewIntroduction) was used to present the thalamic nuclei (Supplementary Fig. 1).

### Grey matter volume of the thalamic nuclei

Since the symptoms of patients with BSP and BOD were bilateral and synchronous, we calculated the total volume of left and right hemisphere for each thalamic nucleus. Then, intergroup differences of 15 thalamic nuclei were performed using a general linear model with age, sex and estimated intracranial volume as covariates. The results were corrected for multi-comparisons using false discovery rate (FDR, *P* < 0.05). *Post hoc* analyses were conducted between any two groups.

### Correlation analyses

Since disease duration and JRS in patients with BOD were not normally distributed, Spearman’s correlations were performed between abnormal thalamic nuclear volume and disease duration/JRS. Pearson’s correlations were performed between abnormal thalamic nuclear volume and disease duration/JRS in patients with BSP with age, sex and estimated intracranial volume as covariates. Statistical significance was set as *P* < 0.05.

### Classification based on support sector machines

To test whether these abnormal features and all 15 thalamic nuclear volumes can be used to distinguish BSP patients from HCs, BOD patients from HCs and BSP from BOD patients, we used support vector machines (SVMs) in Python 3. After regressing out covariates of age, sex and estimated intracranial volume, the residual of abnormal thalamic nuclear volume and all 15 thalamic nuclear volumes were minimum–maximum normalized and then used as inputs to SVM. Sigmoid kernel with main parameters (C, gamma and kernel) was optimized using grid research with 10-fold-cross-validation. Finally, leave-one out was used to test each participate, and accuracy, sensitivity, specificity and area under the curve (AUC) were calculated to evaluate the performance of SVM model.

### Causality analysis of structural covariance network

To further investigate causal relationships among abnormal thalamic nuclei, we performed the regional-based Granger causal analyses (GCA) using the Brain Covariance Connectivity Toolkit (BCCT_V1.2, https://github.com/JLhos-fmri/NeuroimageTools). This method has been successfully used to characterize brain information flow in temporal lobe epilepsy,^32^ schizophrenia,^33^ Parkinson’s disease^34^ and Alzheimer’s disease,^35^ using morphometric data ranked by progression information such as disease duration. In our previous study on BSP, we used a causal structural covariance network (caSCN) to identify two pathways derived by the supplementary motor area—one to areas in the cortico–basal ganglia–brainstem motor pathway and the other to cortical regions in the vision–motor integration pathway.^36^ Specifically, the volumes of abnormal thalamic nuclei in patients with BSP were ranked according to JRS and duration, as well as in patients with BSP and with BOD together. These rankings, although not derived from true longitudinal MRIs of the same patients, functioned as a ‘pseudo-time series’ to characterize disease progression. The *Z*-transformed coefficient-based Granger causality (GC) was used to construct networks among regions with age, sex and estimated intracranial volume as covariates. To maintain consistency with thalamic nuclear atrophy, we included only the positive GC values with *P* < 0.05 (corresponding to *Z* > 1.6).

### Statistical analyses

Age and estimated intracranial volume were compared using analyses of variance among three groups after normality testing by using the Shapiro–Wilk test. Disease duration, JRS scores and BoNT times in the two patient groups were compared using Mann–Whitney U-tests. Chi-squared (*χ*^2^) tests were performed for sex comparisons among the three groups and BoNT injections between two patient groups. These analyses were performed using the Statistical Package for the Social Sciences (SPSS) version 25.0 (SPSS Inc., Chicago, IL).

## Results

### Demographic information and clinical characteristics

In the current study, 56 patients with BSP, 20 with BOD and 58 HCs were included in the analysis (Table 1). A difference in age was observed among the three groups. In addition, a difference in disease duration was observed between the two patient groups. No differences in sex were observed among the three groups, and no differences in JRS scores, number of patients receiving BoNT injections and duration of BoNT injections were observed between the two patient groups.

**Table 1:** Demographics and clinical data

	BSP	BOD	HCs	Groups	
	Mean ± SD	Mean ± SD	Mean ± SD	*F*-values	*P*-values
Subjects	56	20	58		
Age (years)	51.750 ± 8.596	57.300 ± 12.096	49.620 ± 6.950	5.991	0.003^a^
Sex (female/male)	35/21	14/6	39/19		0.787^b^
eTIV (mm^3^)	1,406,615.030 ± 194,077.42	1,436,304.814 ± 140,765.250	1,374,356.962 ± 44.05	0.837	0.435^a^
Disease duration (years)	3.276 ± 3.292	6.045 ± 5.238			0.007^c^
BoNT (yes/no)	38/18	17/3			0.141^b^
BoNT times (years)	1.506 ± 2.397	1.533 ± 2.285			0.990^c^
JRS	5.964 ± 1.159	5.550 ± 2.089			0.278^c^
BFMDRS^d^		6.866 ± 2.510			

BOD, blepharospasm-oromandibular dystonia; BoNT, botulinum toxin; BFMDRS, Burke-Fahn-Marsden Dystonia Rating Scale; BSP, blepharospasm; eTIV, estimated total intracranial volume; HCs, healthy controls; JRS, Jankovic Rating Scale; SD, standard deviation.

^a^The difference of age and estimated total intracranial was obtained by ANOVA among three groups.

^b^Represents *χ*^2^ test.

^c^Represents Mann–Whitney U-test between BSP and BOD groups.

^d^Only 15 subjects were available.

### Thalamic nuclear segmentation

**Figure 1:**
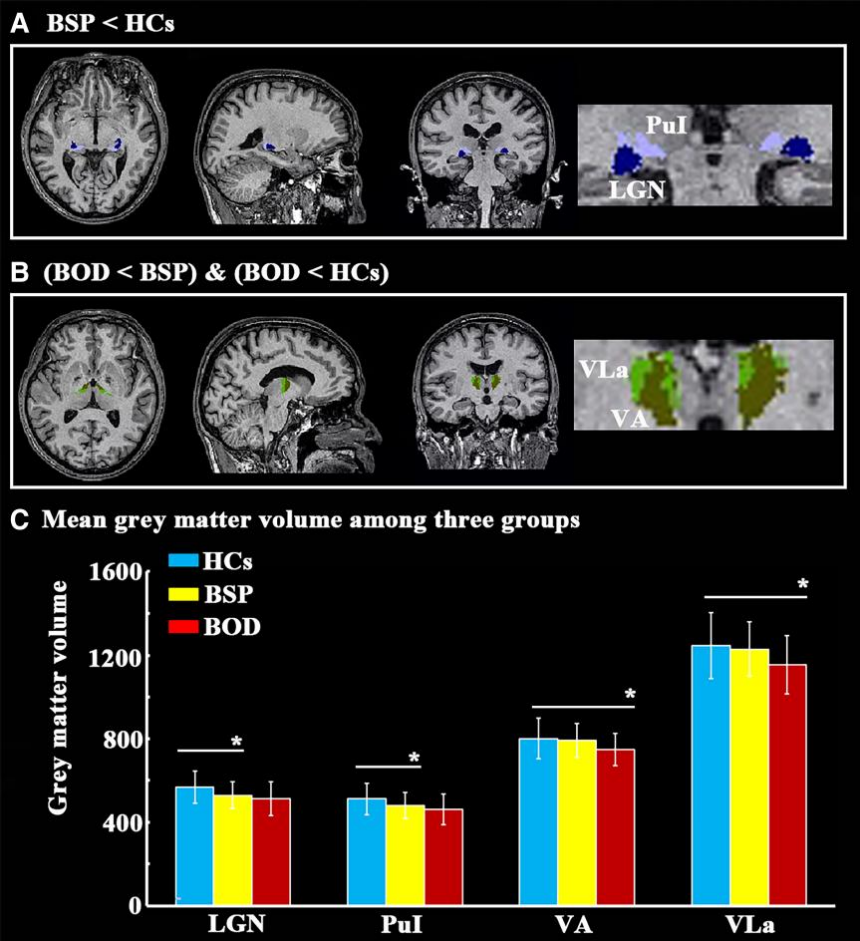
**Differences in thalamic nuclear grey matter volume among the groups.** The thalamic nuclei in patients with BSP showed decreased grey matter volume compared with those in HCs (**A**), comparison between patients with BOD and HCs and comparison between patients with BOD and those with BSP (**B**). (**C**) The difference in mean volume among the three groups was obtained using a general linear model with age, sex and estimated total intracranial as covariates and subsequent *post hoc* two-sample *t*-test analyses for any two groups. *P < 0.05. BOD, blepharospasm-oromandibular dystonia; BSP, blepharospasm; HCs, healthy controls; LGN, lateral geniculate nucleus; PuI, pulvinar inferior nucleus; VA, ventral anterior nucleus; VLa, ventral lateral anterior nucleus.

To further investigate which thalamic nuclei were abnormal in patients with BSP and BOD, we performed thalamic nuclear segmentation. Compared with that in HCs, volumes in the lateral geniculate and pulvinar inferior nuclei in patients with BSP and ventral anterior and ventral lateral anterior nuclei in patients with BOD were significantly decreased (Fig. 1 and Table 2).

**Table 2:** Differences of thalamic nuclear volume among the three groups

	HCs Mean ± SD	BSP Mean ± SD	BOD Mean ± SD	FDR-corrected *P* (ANOVA)	BSP < HCs (*post hoc P*)	BOD < HCs (*post hoc P*)
Lateral geniculate	568.332 ± 76.109	528.952 ± 64.742	513.337 + 79.216	0.048*	0.006*	NS
Pulvinar inferior	511.820 ± 74.143	481.546 ± 62.455	462.254 + 73.892	0.048*	0.024*	NS
Ventral anterior	800.436 ± 95.730	792.388 ± 80.695	747.031 + 77.586	0.048*	NS	0.008*
Ventral lateral anterior	1245.411 ± 156.872	1229.501 ± 130.129	1154.202 + 139.041	0.048*	NS	0.010*

Difference of thalamic nuclear volume among three groups was obtained by using analysis of covariance (ANOVA) with age, sex and estimated total intracranial volume as covariates and subsequent *post hoc* analyses between any two groups. The results were corrected using false discovery rate (FDR). *represents significant results with *P* < 0.05. NS represents *P* > 0.05. BOD, blepharospasm-oromandibular dystonia; BSP, blepharospasm; HCs, healthy controls; SD, standard deviation.

### Correlational analyses

**Figure 2:**
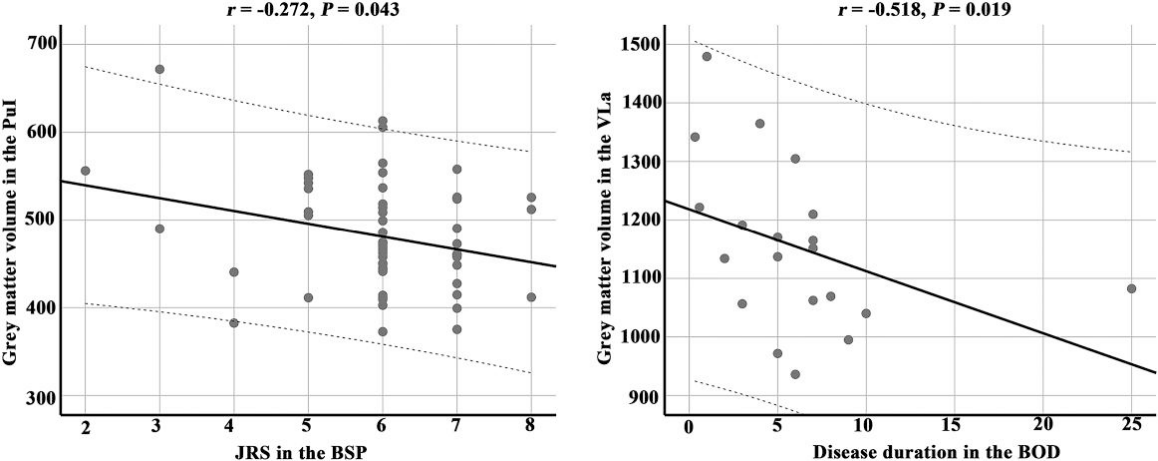
**Correlation results.** Spearman rank correlational analyses indicated a significantly negative correlation between the pulvinar inferior nuclear volume and JRS in patients with BSP (*n* = 58, *r* = −0.272, *P* = 0.043). The ventral lateral anterior nuclear volume negatively correlated with disease duration in patients with BOD (*n* = 20, *r* = −0.518, *P* = 0.019). BOD, blepharospasm-oromandibular dystonia; BSP, blepharospasm; JRS, Jankovic Rating Scale; PuI, pulvinar inferior nucleus; VLa, ventral lateral anterior nucleus.

Spearman rank correlational analyses indicated a significantly negative correlation between the pulvinar inferior nuclear volume and JRS in patients with BSP (*n* = 58, *r* = −0.272, *P* = 0.043). The ventral lateral anterior nuclear volume was negatively correlated with disease duration in patients with BOD (*n* = 20, *r* = −0.518, *P* = 0.019; Fig. 2). To exclude the possibility that this negative correlation may be influenced by a single patient with exceptionally longer disease duration, we excluded this patient and re-analysed the correlation with 19 patients with BOD. The results were similar, with *r* = −0.518 and *P* = 0.023 (Supplementary Fig. 2).

### Classification

**Figure 3:**
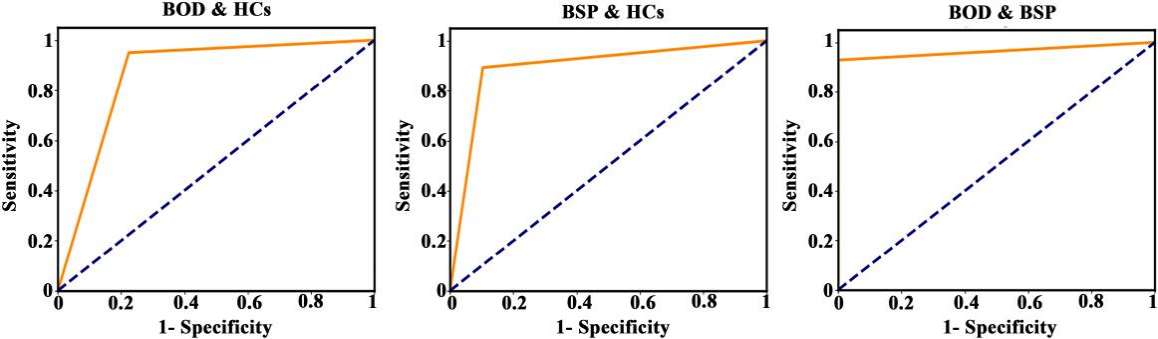
**Results of classification.** Using the grey matter volume of the lateral geniculate, pulvinar inferior, ventral anterior and ventral lateral anterior nuclei as input features, the SVM accurately distinguished BSP patients from HCs (accuracy = 0.89, specificity = 0.89, sensitivity = 0.89 and AUC = 0.89), BOD patients from HCs (accuracy = 0.82, specificity = 0.77, sensitivity = 0.95 and AUC = 0.86) and BSP from BOD patients (accuracy = 0.94, specificity = 1, sensitivity = 0.92 and AUC = 0.96). BOD, blepharospasm-oromandibular dystonia; BSP, blepharospasm; HCs, healthy controls.

Using grey matter volume of the lateral geniculate, pulvinar inferior, ventral anterior and ventral lateral anterior nuclei as input features, the SVM could accurately distinguish BSP patients from HCs (accuracy = 0.89, specificity = 0.89, sensitivity = 0.89 and AUC = 0.89), BOD patients from HCs (accuracy = 0.82, specificity = 0.77, sensitivity = 0.95 and AUC = 0.86) and BSP from BOD patients (accuracy = 0.94, specificity = 1, sensitivity = 0.92 and AUC = 0.96; Fig. 3).

Using the grey matter volume of all 15 thalamic nuclei as input features, the SVM could accurately distinguish BSP patients from HCs (AUC = 0.92), BOD patients from HCs (AUC = 1) and BSP from BOD patients (AUC = 0.97; Supplementary Fig. 3).

### Results of causality analysis of structural covariance network

**Figure 4:**
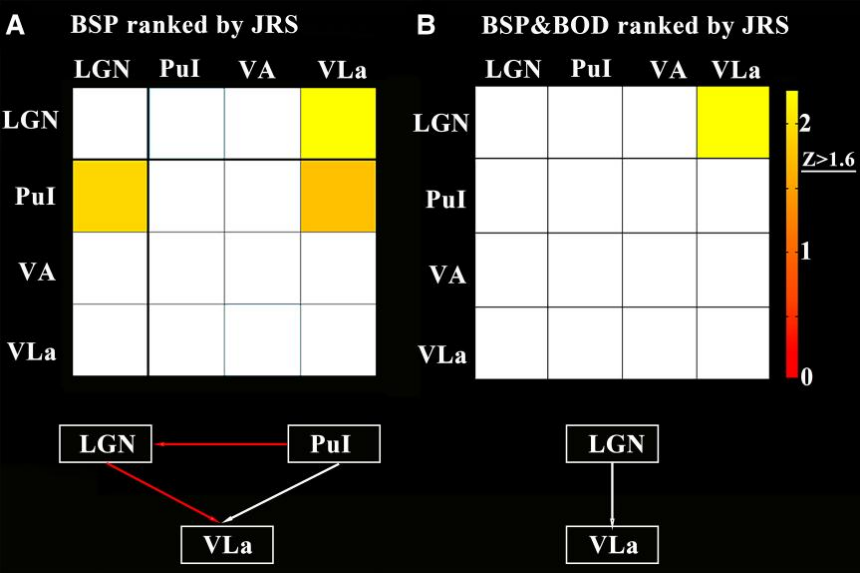
**Causality analysis of structural covariance network.** (**A**) The lateral geniculate nucleus and pulvinar inferior nucleus show significant positive GC with ventral lateral anterior nucleus in the BSP with increasing JRS; pulvinar inferior nucleus shows significant positive GC with the lateral geniculate nucleus. (**B**) The lateral geniculate nucleus shows significant positive GC with ventral lateral anterior nucleus with increasing JRS in the BSP and BOD together. BOD, blepharospasm-oromandibular dystonia; BSP, blepharospasm; JRS, Jankovic Rating Scale; LGN; lateral geniculate nucleus; PuI, pulvinar inferior nucleus; VA, ventral anterior nucleus; VLa, ventral lateral anterior nucleus.

Regional-based GCA was used to construct a network showing an interregional causal relationship among the abnormal thalamic nuclei for BSP, as well as for BSP and BOD together (Fig. 4). Particularly, pulvinar inferior nucleus showed significantly positive GC to the lateral geniculate nucleus and ventral lateral anterior nucleus; lateral geniculate nucleus showed significantly positive GC to the ventral lateral anterior nucleus in BSP patients with increasing JRS; and the lateral geniculate nucleus showed significantly positive GC with the ventral lateral anterior nucleus in patients with BSP and BOD together with increasing JRS.

## Discussion

In the current study, we identified grey matter atrophy in the lateral geniculate and pulvinar inferior nuclei in patients with BSP and in the ventral anterior and ventral lateral anterior nuclei in patients with BOD. Atrophy in the pulvinar inferior nucleus in BSP patients and in the ventral lateral anterior nucleus in BOD patients was negatively correlated with clinical severity and disease duration, respectively. These patterns of thalamic nuclear atrophy could be used as biomarkers to distinguish BSP from BOD patients and those with dystonia from HCs. In addition, GCA revealed that pulvinar inferior nuclear atrophy extended to lateral geniculate nuclear atrophy and then to ventral lateral anterior nuclear atrophy with increased clinical severity in patients with BSP.

Specifically, we identified grey matter atrophy in the lateral geniculate and pulvinar inferior nuclei in patients with BSP. The lateral geniculate and pulvinar inferior nuclei are both considered as the subcortical visual relay centres of visual pathways connecting the retina and superior colliculus.^37^ They share the same koniocellular visual pathway^27^ and play similar roles in synchronizing oscillatory activity of thalamo-cortical loops and modulating visual cortical activation.^38^ The lateral geniculate nucleus accepts sensory input from the retina, which is mainly involved in visual perception.^39^ Sensory symptoms, such as a burning sensation, grittiness, dry eye and photophobia,^40^ are commonly reported in patients with BSP. Previous studies also indicated that photophobia may be associated with abnormal activity in lateral geniculate nucleus.^41-43^ Moreover, one study suggested that the lateral geniculate nucleus might play an important role in visual eyeblink conditioning by supplying visual sensory input to the pontine nuclei and receiving feedback from the cerebellum.^42^ It is possible that these symptoms might be caused by lateral geniculate nuclear dysfunction in BSP.

The pulvinar inferior nucleus is considered as a sensory link for visual signals sent from the superior colliculus to the cortex,^44^ which are mainly involved in the control of visual attention and oculomotor behaviour.^45^ Previous animal studies revealed synchronized activity between the pulvinar inferior nucleus and cortical areas during attention allocation, suggesting a critical role in regulating information transmission across the visual cortex.^46^ Moreover, the pulvinar inferior nucleus is thought to be important for the initiation and compensation of saccadic eye movement.^47^ Several previous studies on eye movements in patients with BSP demonstrated longer latencies but normal peak velocities and gains in the eye saccades.^47^ In addition, our correlation analysis showed negative correlation between pulvinar inferior nuclear volume and JRS scores in the BSP. The pulvinar inferior nuclear dysfunction might be associated with longer saccadic latency in BSP. However, caution is warranted, as no direct correlation between the measurement of longer saccadic latency and grey matter volume of the pulvinar inferior nucleus was identified in the present study. Our GCA results also showed significant causal relationship between pulvinar inferior nuclear atrophy and lateral geniculate nuclear atrophy in the BSP. It is reasonable to speculate that the grey matter atrophy in the pulvinar inferior nucleus occurred prior to that in the lateral geniculate nucleus.

We also identified grey matter atrophy in the ventral lateral nucleus and ventral anterior nucleus in patients with BOD compared with that in HCs. These atrophied regions are known as the motor thalamus, connecting the motor areas of the cerebral cortex to the basal ganglia and cerebellum.^48^ Furthermore, proprioceptive signals from muscles have historically been considered to project to the ventral posterolateral nucleus.^49^ Ventral lateral and ventral anterior nuclear atrophy may therefore account for the widely spread movement dysfunction of the lower facial muscles, mouth, jaw, tongue and pharyngeal and cervical muscles in the BOD.^27^ In addition to controlling limb movements, the ventral lateral nucleus also played unique functional roles in jaw movement motor control in rats^50^ and guinea pigs.^51^ Because jaw movement dysfunction represents the earliest and most common spread symptoms in the BOD,^52,53^ we speculated that the ventral lateral nuclear atrophy might be associated with jaw movement dysfunction. Our correlation results showing negative correlation between ventral anterior nuclear atrophy and disease duration also support this speculation.

Most importantly, our GCA analyses further revealed that lateral geniculate nuclear atrophy extended to ventral lateral anterior nuclear atrophy with increasing JRS in patients with BSP, as well as in those with BSP and BOD together. We speculated that the pulvinar inferior nuclear atrophy might be the focal origin of BSP causing eye movement symptoms, extending to the lateral geniculate causing visual sensory symptoms in the BSP and subsequently extending to the ventral lateral anterior nuclei resulting in involuntary lower facial and masticatory movements known as BOD. It is also consistent with the sequential order of the symptoms that occurred in patients with BSP and BOD.^1^ However, caution needs to be paid because these atrophy patterns in the thalamic nuclei were based on ‘pseudo-time series’ according to JRS score rather than true time series from longitudinal analyses.

These results also suggested that ventral lateral anterior nuclear atrophy might be a neurobiomarker of transformation from BSP to BOD and could be used as a potential target for deep brain stimulation (DBS). As it is known, the majority of DBS in patients with BOD were performed at the target of the globus pallidus internus (GPi) or subthalamic nucleus (STN).^54^ Although they yielded markedly improved symptoms with an average decreased BFMDRS score of 61–66% at 1 year, and sustained score after 3 years,^55^ 10–20% of patients showed improvement below 25–30% and even no response to these DBS treatments.^56^ In addition, side effects, such as stimulation-induced dyskinaesia, cognition and other non-motor symptoms, may occur in some patients, adversely affecting surgical outcomes and patient satisfaction.^57^ Therefore, it is necessary to identify a novel DBS target with fewer side effects and is efficacious for patients who were not optimally responsive to current DBS targets. Indeed, a previous case report showed a patient with DYT6 dystonia whose BFMDRS total score decreased from 71 to 11 at the 2-year follow-up after continuous bilateral thalamic ventral lateral anterior nucleus DBS.^58^ Another case report also showed that the patient achieved a significant benefit at the 3-year follow-up postoperatively and continued to experience strong benefit and improvement of dystonia symptoms with minimal adverse effects from bilateral DBS implantation in the alternative targets of the ventralis oralis anterior and posterior nuclei of the thalamus.^59^ Although our results might serve as a fundamental explanation of the success of these case reports, further studies with large sample size are still needed to verify whether the ventral lateral anterior nucleus could be used as effective and alternative DBS target for BOD.

Despite the interesting findings, there are several major limitations that need to be stressed. First, sample size of BOD group is relatively small because only ∼50% of the BSP cases experience spread to other parts of the body.^60^ A previous study reported that only 38.7% of BSP patients progress to BOD.^27^ This ratio is comparable to that observed in our current study. However, a larger sample size of BOD is required to confirm our findings. Second, the average age of BOD group was greater than that of the BSP group. This is consistent with a previous study, which found that the average age of BOD dystonia onset was ∼5 years greater than that of BSP.^27^ Although we included age as a covariate in our general linear models and GCA, we cannot fully exclude the potential effects of age on our results. Third, we only used high-resolution T_1_ images with a voxel size of 1 mm × 1 mm × 1 mm in the thalamic nuclear segmentation. Additional MR images, such as fast grey matter acquisition T_1_ inversion recovery, might be useful to improve the accuracy thalamic nuclear segmentation, especially for small nuclei. Therefore, we excluded 10 thalamic nuclei as they are relatively small with mean grey matter volume < 200 mm^3^. Finally, it is a cross-sectional study, and a longitudinal design is required to verify our results, especially for the GCA results, which were obtained from ‘pseudo-time series’ according to the disease progression.

Taken together, these results suggest that the pulvinar inferior nuclear atrophy might be the focal origin of BSP, extending to lateral geniculate nuclear atrophy and subsequently extending to ventral lateral anterior nucleuscausing involuntary lower facial and masticatory movements known as BOD. Moreover, our results also provide potential targets for neuromodulation especially DBS in patients with BOD.

## Supplementary Material

fcae117_Supplementary_Data

## Data Availability

Data are available from the corresponding authors upon request. FreeSurfer (https://surfer.nmr.mgh.harvard.edu/fswiki) was used for thalamic nuclei segmentation. The Brain Covariance Connectivity Toolkit (BCCT_V1.2, https://github.com/JLhos-fmri/NeuroimageTools) was used to investigate causal relationships among abnormal thalamic nuclei. The SVM in Python 3 was used for classification. The SPSS 25 (SPSS Inc., Chicago, IL; https://spss.en.softonic.com/) was used for statistical analyses.
