# Supplementary material for: Progressive thalamic nuclear atrophy in blepharospasm and blepharospasm-oromandibular dystonia
Source: Brain Commun. 2024 Apr 8;6(2):fcae117. doi: 10.1093/braincomms/fcae117 (PMC11025674; doi:10.1093/braincomms/fcae117)
Supplement: fcae117_Supplementary_Data [file fcae117_supplementary_data.zip › Supplementary_figure_legends.docx]

**Supplementary Figure legends**

**Supplementary Figure 1**. **Thalamic nucleus of one healthy participant.** The images were shown using Freeview (https://surfer.nmr.mgh.harvard.edu/fswiki/FreeviewGuide). Thalamic nuclei with * were not involved in further analysis as they are relatively small with mean gray matter volume less than 200.

**Supplementary Figure 2. Correlation analysis.** To exclude the possibility that this negative correlation may be driven by one single patient with an exceptionally longer disease duration, we exclude this patient and re-analyzed the correlation with 19 patients with BOD. The ventral lateral anterior nuclear volume was also negatively correlated with disease duration in patients with BOD (n = 19, *r* = -0.518, *P* = 0.023). Abbreviations: BOD, blepharospasm-oromandibular dystonia; and VLa, ventral lateral anterior nucleus.

**Supplementary Figure 3. Results of classification.** Using the gray matter volume of all 15 thalamic nuclei as input features, the SVM could accurately distinguish BSP patients from HCs (AUC = 0.92), BOD patients from HCs (AUC = 1), and BSP from BOD patients (AUC = 0.97). Abbreviations: BOD, blepharospasm-oromandibular dystonia; BSP, blepharospasm; and HCs, healthy controls.
