# Supplementary figures and images for: Progressive thalamic nuclear atrophy in blepharospasm and blepharospasm-oromandibular dystonia
Source: Brain Commun. 2024 Apr 8;6(2):fcae117. doi: 10.1093/braincomms/fcae117 (PMC11025674; doi:10.1093/braincomms/fcae117)

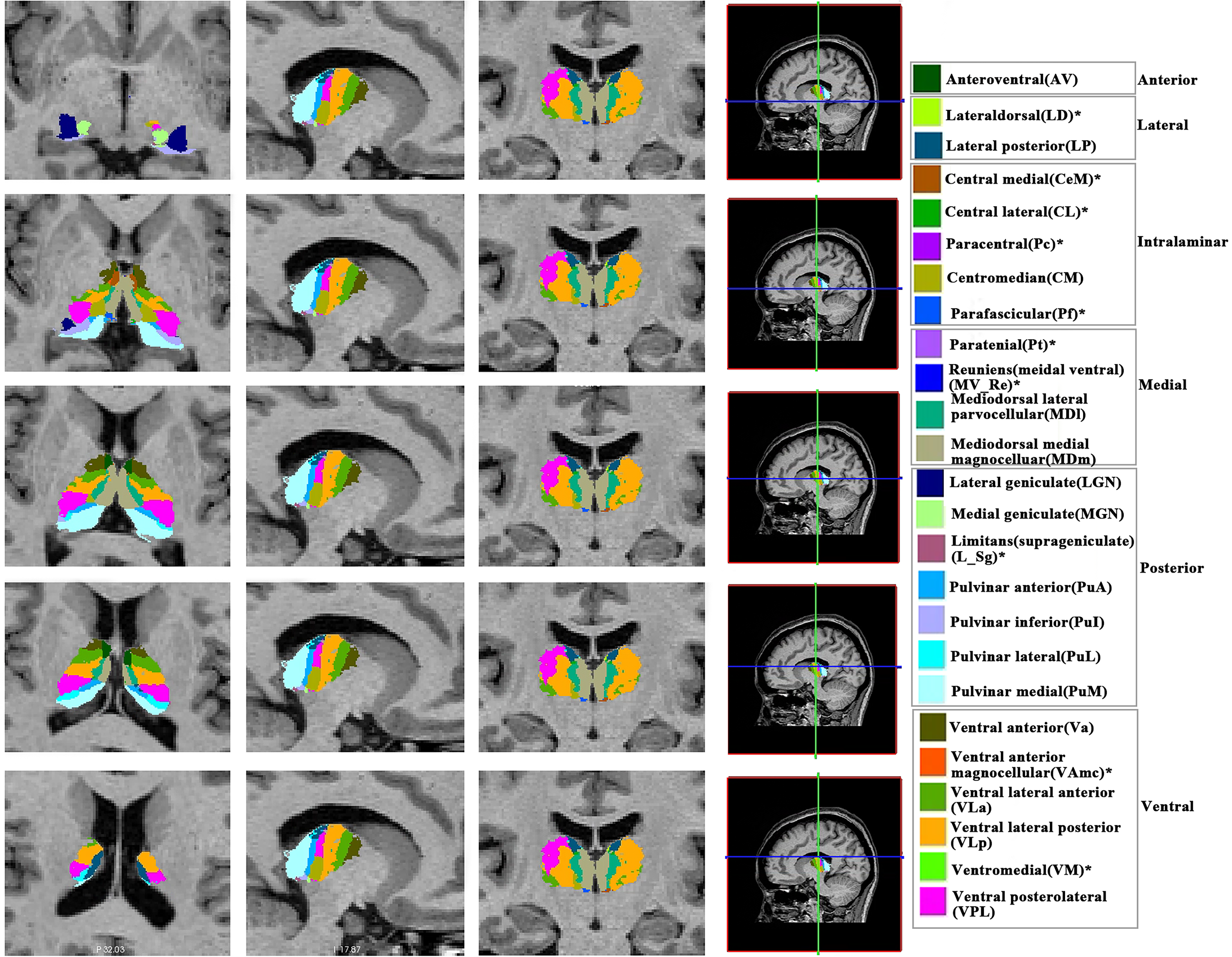

Supplement: fcae117_Supplementary_Data [file fcae117_supplementary_data.zip › Supplementary Figure 1.tif]

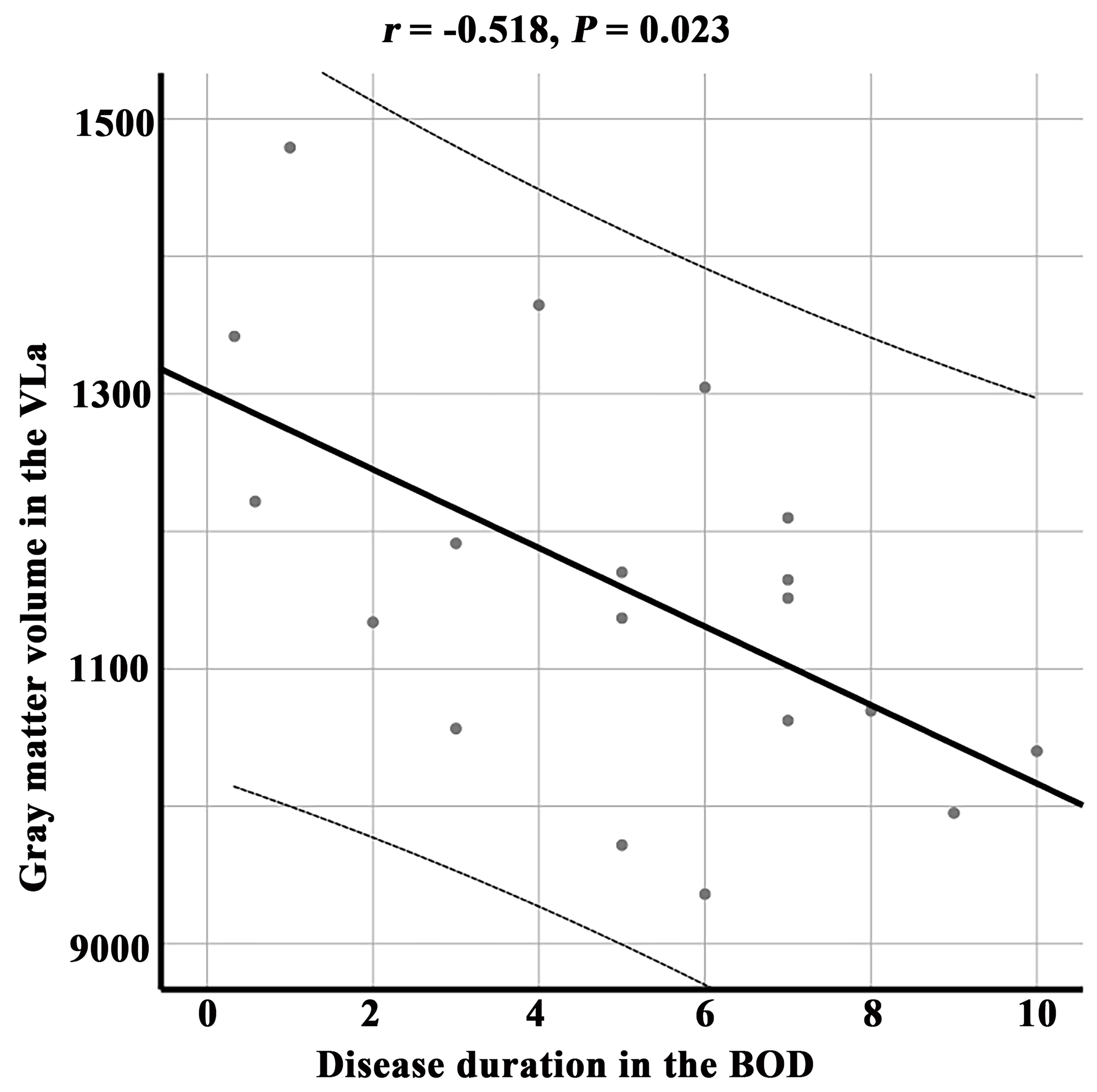

Supplement: fcae117_Supplementary_Data [file fcae117_supplementary_data.zip › Supplementary Figure 2.tif]

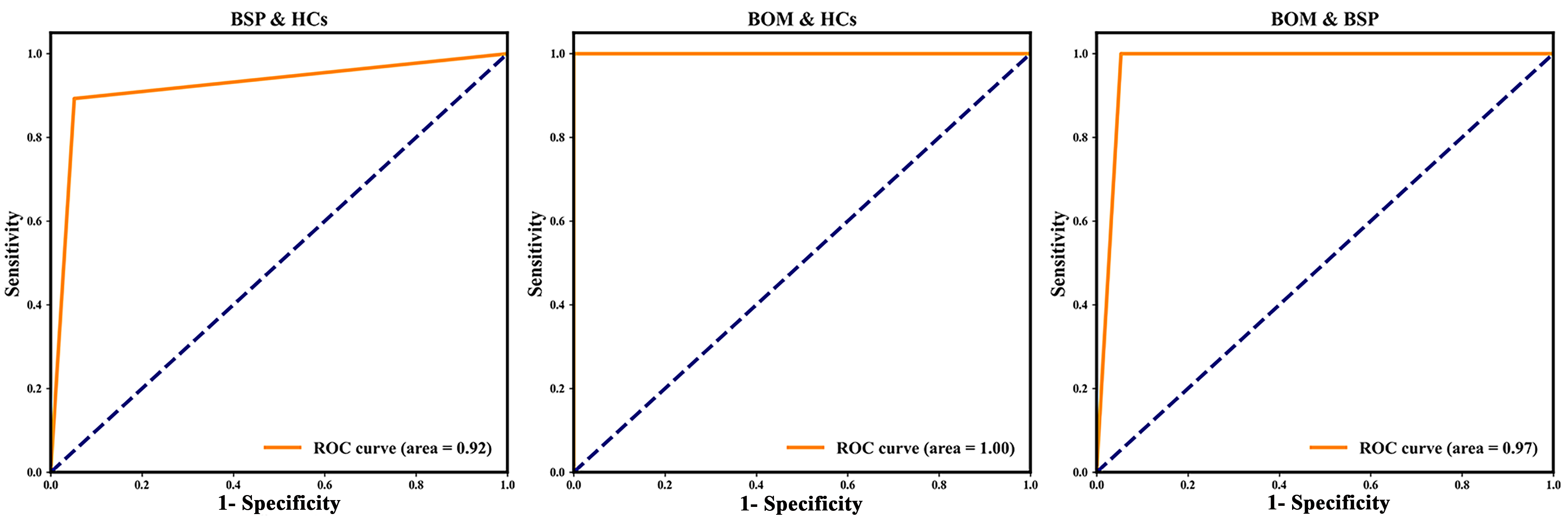

Supplement: fcae117_Supplementary_Data [file fcae117_supplementary_data.zip › Supplementary Figure 3.tif]
